# Chronic administration of isocarbophos induces vascular cognitive impairment in rats

**DOI:** 10.1111/jcmm.12775

**Published:** 2016-01-28

**Authors:** Peng Li, Ya‐Ling Yin, Mo‐Li Zhu, Guo‐Pin Pan, Fan‐Rong Zhao, Jun‐Xiu Lu, Zhan Liu, Shuang‐Xi Wang, Chang‐Ping Hu

**Affiliations:** ^1^ Department of Pharmacology Pharmaceutical College Central South University Changsha China; ^2^ College of Pharmacy Xinxiang Medical University Xinxiang China; ^3^ College of Basic Medical Sciences Tongji Medical School Huazhong University of Science and Technology Wuhan China; ^4^ School of Basic Medical Sciences Xinxiang Medical University Xinxiang China; ^5^ Sanqun Medical College Xinxiang Medical University Xinxiang China; ^6^ Department of Clinical Nutrition The Affiliated Hospital Hunan Normal University Changsha China; ^7^ The Key Laboratory of Cardiovascular Remodeling and Function Research Qilu Hospital Shandong University Jinan China

**Keywords:** vascular cognitive impairment, organophosphorus, endothelial dysfunction, ageing

## Abstract

Vascular dementia, being the most severe form of vascular cognitive impairment (VCI), is caused by cerebrovascular disease. Whether organophosphorus causes VCI remains unknown. Isocarbophos (0.5 mg/kg per 2 days) was intragastrically administrated to rats for 16 weeks. The structure and function of cerebral arteries were assayed. The learning and memory were evaluated by serial tests of step‐down, step‐through and morris water maze. Long‐term administration of isocarbophos reduced the hippocampal acetylcholinesterase (AChE) activity and acetylcholine (ACh) content but did not alter the plasma AChE activity, and significantly damaged the functions of learning and memory. Moreover, isocarbophos remarkably induced endothelial dysfunction in the middle cerebral artery and the expressions of ICAM‐1 and VCAM‐1 in the posterior cerebral artery. Morphological analysis by light microscopy and electron microscopy indicated disruptions of the hippocampus and vascular wall in the cerebral arteries from isocarbophos‐treated rats. Treatment of isocarbophos injured primary neuronal and astroglial cells isolated from rats. Correlation analysis demonstrated that there was a high correlation between vascular function of cerebral artery and hippocampal AChE activity or ACh content in rats. In conclusion, chronic administration of isocarbophos induces impairments of memory and learning, which is possibly related to cerebral vascular dysfunction.

## Introduction

Vascular dementia, being the most severe form of vascular cognitive impairment (VCI), is present in at least 20% of cases of dementia caused by cerebrovascular disease, secondary to Alzheimer's disease (AD) 1. Vascular cognitive impairment typically develops when the blood supply to the brain is reduced or blocked by a damaged vascular system with advanced age or other risk factors 2. A chronic and continuous reduction in cerebral blood flow affects neural structures regulating memory and cognitive processes and eventually leads to the formation of dementia 3.

Organophosphate pesticides, used extensively in agriculture, have the potential to produce several forms of toxicity as a result of inhibition of plasma acetylcholinesterase (AChE) activity 4. Organophosphorus increased acetylcholine (ACh) in cholinergic synapses in the peripheral neuron system and results in the overstimulation of nicotinic and muscarinic receptors, which is secondary to AChE inhibition 5, 6. Moreover, organophosphorus produces a very specific syndrome of delayed neuron disorders without the suppression of plasma AChE activities 7. The detrimental effects of chronic exposure or accumulation of organophosphorus have been investigated by several studies. For example, chronic administration of lower doses of malathion, fenitrothion or acephate shows increased blood glucose in adult rats 8. Prenatal exposure of guinea pig to chlorpyrifos disrupts the structural and functional integrity of the brain 9. However, the effects of long‐term exposure of organophosphorus on VCI are not well known.

Recently, long‐term administration of organophosphorus caused vascular dysfunction, accelerated the formation of atherosclerosis and other diseases related to cardiovascular diseases 8, 10, 11. Evidence from epidemiology indicate that chronic organophosphorus exposure would lead to the impairments of neurocognitive functions 12, 13, 14, 15. Thus, we suggested that chronic organophosphorus exposure might induce cerebrovascular dysfunctions and sequential cognitive impairments. Our results indicated that chronic exposure to 16 weeks of administration of isocarbophos caused vascular dysfunction in rat cerebral artery and impairments of learning and memory in hippocampus.

## Materials and methods

A full description of materials, methods and research design can be found in the online‐only Data S1.

## Results

### Isocarbophos reduces the latency and increases error numbers in step‐down avoidance test

To test whether organophosphor is a risk factor for vascular dementia, the model of VCI in SD rats were established by treating with isocarbophos (0.5 mg/kg per 2 days), which is widely used in developing countries, and even in developed countries 16, 17. Model of the permanent ligation of bilateral common carotid artery occlusion (BCCAO) was used as positive control of vascular dementia, the most severe form of VCI. Plasma AChE activity was assayed to determine the chronic poisoning effects of isocarbophos. In Table S1, plasma AChE activities were identical in each group during the whole experiments (the 4th–16th week), indicating that the chronic administration of isocarbophos has no inhibitory effects on the plasma AChE activity, which is different to acute organophosphor poisoning as it decreases plasma AChE activity 18.

**Figure 1:**
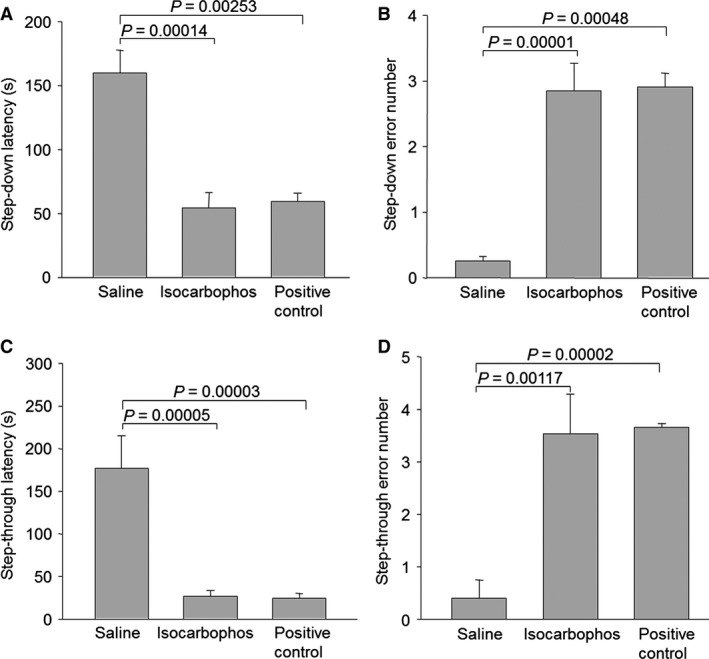
Isocarbophos reduces the latency and increases error numbers in step‐down test and step‐through test in rats. After the end of experiment, (**A**) the latency and (**B**) error numbers in step‐down test, and (**C**) the latency and (**D**) error numbers in step‐through test were recorded and analysed. Data are reported as means ± S.E. *N* is 6 in each group. All results were analysed using a one‐way anova followed by Newman–Student's *t*‐test.

The short‐term memory was evaluated by step‐down test at the 16th week after isocarbophos treatment. As shown in Figure 1A, compared to saline‐treated rats, the latency of step‐down test in positive control rats was much shorter, indicating that the model of vascular dementia has been successfully established. However, treatment with isocarbophos dramatically reduced the latency time. Besides, analysis of error number indicated that it was much higher in isocarbophos‐treated rats or positive control rats, compared to the saline gavage rats (Fig. 1B). These data suggest that the long‐term administration of isocarbophos at a dose of 0.5 mg/kg every 2 days, similar to BCCAO, damages short‐term memory in rats.

### Isocarbophos decreases the latency and increases error numbers in passive avoidance step‐through task test

The isocarbophos‐induced impairment on short‐term memory in rat was further confirmed by passive avoidance step‐through task test. In the 16th week, the latency of step‐through test in isocarbophos‐treated rats or positive control rats was significantly lower than saline‐treated rats (Fig. 1C). Moreover, administration of isocarbophos increased error number in this test (Fig. 1D). These evidence also further support our notion.

### Isocarbophos impairs spatial memory in morris water maze test

Besides short‐term memory, the spatial memory was also evaluated by morris water maze test for learning and memory behaviour assessment 19. As shown in Table S2, swimming distance in quadrant, ratio of swimming distance in quadrant to total swimming distance, swimming time in quadrant, swimming time in quadrant to total swimming time and number of escape were increased in isocarbophos‐treated rats and positive control rats. Conversely, the swimming time and the distance out of quadrant were increased in isocarbophos‐treated rats and positive control rats. This finding indicates that long‐term administration of isocarbophos, as well as BCCAO, causes spatial learning and memory impairment.

By analysing the swimming distance, swimming speed in quadrant and the average of swimming speed (Table S2), we found that there was no significant difference between any two groups, demonstrating that motor function was not impaired by isocarbophos.

### Isocarbophos has no effect on nutrient metabolism and liver functions

Long‐term exposure to isocarbophos through gavage may affect the nutrient metabolism and damage liver functions, which may contribute to the behavioural abnormalities in rats. Thus, we performed blood biochemical analysis and detected liver functions. As shown in Table S3, isocarbophos at the dose of 0.5 mg/kg per 2 days for 16 weeks of administration did not alter the levels of plasma K^+^, Na^+^, Cl^−^, Ca^2+^, Mg^2+^, blood urine nitrogen, creatinine, glucose, triglycerin and cholesterol, except the light enhancement of homocysteine. The liver functions, such as glutamic‐pyruvic transaminase, glutamic‐oxalacetic transaminase, total protein, albumin, globulin, total bilirubin, direct bilirubin and indirect bilirubin were comparable in rats treated with saline and isocarbophos. These data indicate that isocarbophos‐induced cognitive impairment is not because of hepatic and metabolic encephalopathy in rats.

### Isocarbophos disorders the structure of rat hippocampal neurons

**Figure 2:**
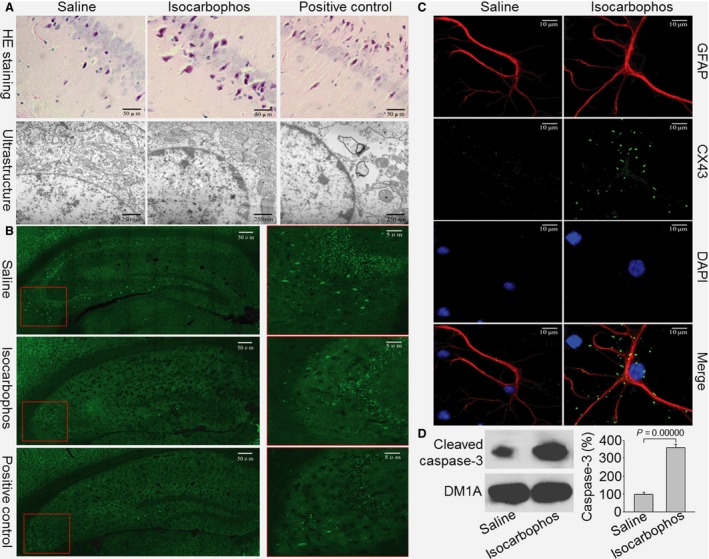
Isocarbophos disorganizes structure of hippocampus in rats. (**A** and **B**) After the experiment, hippocampus tissue was isolated from rat. (**A**) Morphological analysis was performed under light microscope by haematoxylin and eosin staining (400×) or ultrastructure by electron microscope. (**B**) The cholinergic neurons in hippocampus were detected by IFC. Green colour indicates cholinergic neurons. (**C**) Cultured rat primary astroglial cells were treated with isocarbophos (25 μM) for 48 hrs. CX43 was assayed by IFC. GFAP, Cytosol marker. DAPI, Nucleus marker. All pictures are a representative picture from six repeats. (**D**) Cultured primary hippocampal neurons were treated with isocarbophos (25 μM) for 48 hrs. Total cell lysates were subjected to perform western blotting analysis of cleaved caspase‐3. DM1A serves as a loading control. Data are expressed as means ± S.E. *N* is 5 in each group. An unpaired Student's *t*‐test was used for statistical analysis.

The intact hippocampus is necessary for the acquisition of spatial information 20, 21. We next examined the morphology of hippocampal neurons in rats at the end of experiments by haematoxylin and eosin staining and electron microscopy. Treatment of isocarbophos provoked many histological changes in the hippocampus, such as the disappearance of neurons and shrunken neurons with darkly stained condensed nuclei (Fig. 2A). Pyramidal cells in three layers arranged in disorder and partially swelled, accompanied by the infiltration of inflammatory cells and the proliferation of astroglia cells. Some red blood cells and angiogenesis were also seen in isocarbophos‐treated rat hippocampus. All these alterations were positive in BACCO rats, but not in saline‐treated rats.

We further used electron microscope to observe the structure of hippocampal neurons in rat. As shown in Figure 2A, the inner membrane of hippocampal blood–brain barrier in isocarbophos‐treated rat was not smooth. The neurons were smaller and the membrane structures were unclear with shrank nucleus and the aggregated chromatin to the edges. Increased number of Golgi body, swelled and infused mitochondrial appeared in hippocampus from rats treated with isocarbophos, consistent with morphological analysis under light microscopy.

### Isocarbophos reduces the number of cholinergic neurons and damages neurons and astroglial cells in rat hippocampus

The detrimental effects of isocarbophos on the hippocampus were further confirmed by detecting the numbers of cholinergic neurons in rat hippocampus. As depicted in Figure 2B, the 16‐week treatment of isocarbophos or BACCO dramatically decreased the numbers of cholinergic neurons indicated by staining with choline acetyltransferase (ChAT) as a biomarker of cholinergic neuron 22.

The damages of astroglial cells and hippocampus neurons by isocarbophos were determined by detecting the levels of CX43 and cleaved caspase‐3 in cultured primary cells. After 48 hrs of treatment, isocarbophos remarkably increased CX43 levels in astroglial cells (Fig. 2C) and cleaved caspase‐3 levels in hippocampus neurons (Fig. 2D).

### Isocarbophos reduces both AChE activity and ACh content in hippocampal neurons

**Figure 3:**
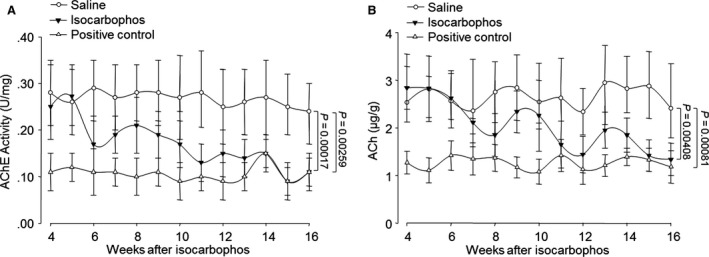
Isocarbophos inhibits AChE activity and decreases ACh content in hippocampal tissue from rats. Homogenate of rat hippocampal tissue was subjected to detect (**A**) AChE activity and (**B**) ACh content. AChE, acetylcholinesterase. ACh, acetylcholine. Data are expressed as means ± S.E. *N* is 6 in each group. All results were analysed using a two‐way anova followed by Newman–Student's *t*‐test.

The central cholinergic system plays important roles in the regulation of cerebral circulation and cognitive function 23. Thus, we investigated whether isocarbophos alters the AChE activity and ACh content in the hippocampus of rats. As shown in Figure 3A and B, both the AChE activity and ACh content were time‐dependently decreased in the hippocampus from isocarbophos‐treated rats. In positive control group, both AChE activity and ACh content in the hippocampus were decreased after surgery and kept in low level during the whole experiments, consistent with previous studies 19. Together, it reveals that isocarbophos might damage hippocampal cholinergic neurons to induce cognitive impairment in rats.

### Isocarbophos impairs endothelium‐dependent relaxation in cerebral artery

**Figure 4:**
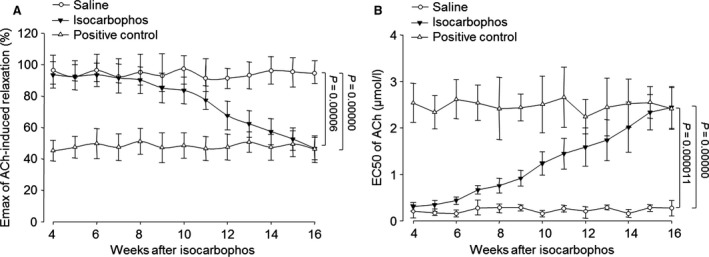
Isocarbophos time‐dependently impairs endothelium‐ dependent relaxation in middle cerebral artery in rats. ACh‐induced endothelium‐dependent relaxation of isolated middle cerebral artery was assayed by organ chamber in rats. (**A**) Effects of isocarbophos on Emax of ACh‐induced relaxation. (**B**) Effects of isocarbophos on EC50 of ACh‐induced relaxation. Data are reported as means ± S.E. *N* is 6 in each group. All results were analysed using a two‐way anova followed by Newman–Student's *t*‐test.

Vascular cognitive impairment typically develops when blood supply to the brain is reduced or blocked by risk factors 24. To determine whether isocarbophos‐induced dementia is highly related to vascular dysfunction, we examined the vascular relaxation in rat middle cerebral artery by organ chamber. As show in Figure 4A, the maximum effect (Emax) of ACh‐induced vasorelaxation in the middle cerebral artery from isocarbophos‐treated rats gradually decreased beginning at the 8th week after administration of isocarbophos with (0.5 mg/kg per 2 days) and reached the similar levels to positive control rat at the 15th–16th week. Oppositely, EC50 of ACh on middle cerebral artery relaxation was significantly higher in isocarbophos‐treated or positive control rats than in saline‐treated rat (Fig. 4B). Collectively, these data indicate that isocarbophos induces cerebral arterial dysfunction in rats.

### Vascular structure is disorganized by isocarbophos in rat cerebral artery

**Figure 5:**
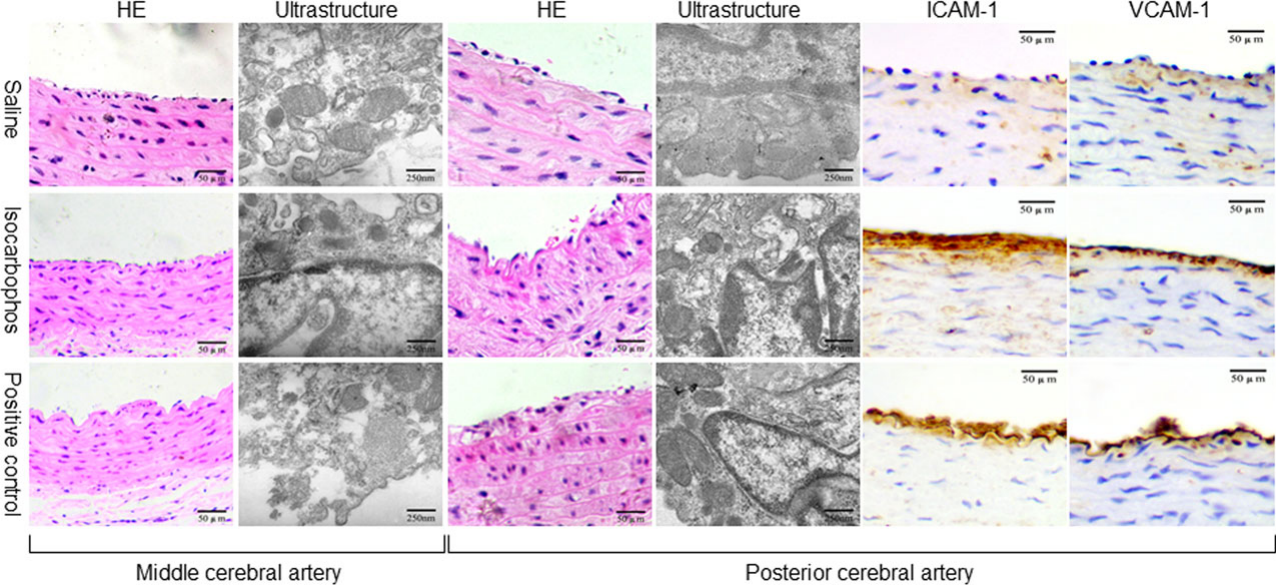
Isocarbophos induces vascular structure abnormalities of middle and posterior cerebral arteries in rats. After the experiment, middle and posterior cerebral arteries were isolated from rats. The morphologies of vascular wall were determined under light microscope by haematoxylin and eosin staining (400×) or ultrastructure by electron microscope. The expressions of ICAM‐1 and VCAM‐1 in posterior cerebral artery were determined by IHC. All pictures are a representative picture from six repeats.

We also analysed the morphology of vascular structure in rat middle and posterior cerebral arteries. As depicted in Figure 5, the intact of endothelium layer, thinness and irregular arrangement of smooth muscle layer and breakdown of elastin in the middle and posterior cerebral arteries were observed in isocarbophos‐treated rats or positive control rats by haematoxylin and eosin staining at the 16th week after isocarbophos treatment. Under electron microscope, disintegrity of endothelial cells membrane, merged smooth muscle cells with endothelial cells, disruptions of blood–brain barrier and swelled and fused mitochondria were visible in middle and posterior cerebral arteries from isocarbophos‐treated rats or positive control rats, but not from saline‐treated rats. These observations support that isocarbophos is detrimental to cerebral arterial remodelling.

### Isocarbophos induces inflammation in rat posterior cerebral artery

Vascular inflammation is an important contributor to vascular dysfunction. We next detected the expressions of VCAM‐1 and ICAM‐1 in posterior cerebral arteries. As presented in Figure 5, the expressions of both VCAM‐1 and ICAM‐1 were significantly increased in posterior cerebral artery from isocarbophos‐treated rats or positive control rats. This indicates that isocarbophos induces vascular inflammation, which may contribute to cerebral arterial dysfunction.

### Isocarbophos‐induced hippocampal damage is related to cerebral arterial dysfunction

**Figure 6:**
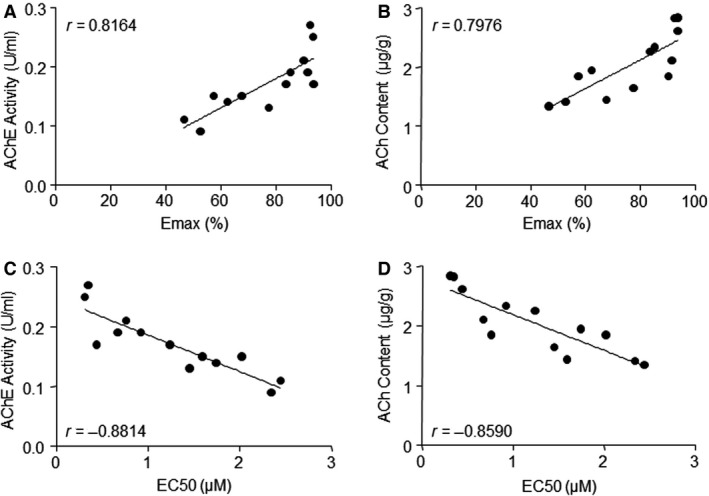
Correlation between vascular function of middle cerebral artery and AChE activity or ACh content in hippocampal tissue from isocarbophos‐treated rats. (**A**) Correlation of Emax and AChE activity. (**B**) Correlation of Emax and ACh content. (**C**) Correlation of EC50 and AChE activity. (**D**) Correlation of EC50 and ACh content. AChE, acetylcholinesterase. ACh, acetylcholine. Emax, maximal effect induced by ACh.

To establish the link of vascular dysfunction with hippocampal damage in isocarbophos‐treated rats, the corrections between ACh‐induced vasorelaxation (Emax and EC50) in the middle cerebral artery and the AChE activity and ACh content in hippocampal cholinergic neurons were analysed. In Figure 6A, the correlation coefficient (*r*‐value) of Emax in middle cerebral artery and AChE activity in the hippocampus of the isocarbophos‐treated group are 0.8164 and 95% confidence interval (CI) is 0.4823–0.9432. Similarly, *r*‐value and 95% CI between Emax and ACh content are 0.7976 and 0.4399–0.9369 (Fig. 6B). The *r*‐value and 95% CI of EC50 in brain artery and AChE activity in hippocampus are 0.8814 and 0.6424–0.9642 (Fig. 6C). There is also a negative correlation between EC50 and ACh content in hippocampus (*r* = −0.8590; 95% CI, 0.5848–0.9570; Fig. 6D). All results indicate that isocarbophos‐caused impairment of learning and memory is possibly related to vascular dysfunction in cerebral artery.

## Discussion

Chronic exposure of organophosphates does not evoke cholinergic symptoms such as lachrymation, salivation, meiosis or muscle fasciculation, which suggests that their negative impact on cognition is unrelated to plasma AChE inhibition 4. Besides, organophosphates also produces the delayed secondary neuronal destruction, which arises primarily in the cholinergic areas of the brain that contain dense accumulations of cholinergic neurons and the majority of cholinergic projection, could be largely responsible for persistent profound neuropsychiatric and neurological impairments, such as AD 25. The major finding in this project is that chronic exposure to isocarbophos damaged the memory, cognition, learning and the structure of hippocampus.

A key issue of this study is how to determine the optimal dose and the exposed time of isocarbophos in rats. It is important to distinguish between such dose effects and the well characterized potential of higher levels of organophosphates exposure to cause an acute cholinergic crisis with lasting secondary consequences. In this study, we justified the dose and exposing time by three points as followings. Firstly, in light of the preliminary experiments and our previous studies 26, we observed isocarbophos at this dose did not inhibit the AChE activity in plasma and did not produce acute cholinergic symptoms. Secondly, by comparing to the dose of chlorpyrifos used by Mullins RJ, it was 20 mg/kg for 10 consecutive days to disrupt the structural and functional integrity of the brain in pig 9. This is much higher and shorter than ours. Thirdly, Rodgers *et al*. observed that purified malathion at the dose of 0.25 mg/kg increased cellular respiratory burst without inhibition of AChE activity in rats 27. This is a very low dose defined by them. Evenly, they reported that oral dose of malathion as low as 0.1 mg/kg was effective to induce mast cell degranulation 28. Thus, we made a reasonable speculation that the dose of isocarbophos used by us is also a low‐dose.

Another important discovery of this study is that isocarbophos induces vascular dysfunction in rat cerebral artery. We and others have previously reported that oral administration of organophosphates impaired vascular endothelial dysfunction in rabbit 26 or subtle microvascular injury in mouse 29. In this present study, we measured the vascular remodelling and function of rat cerebral artery. The results indicate that isocarbophos induces vascular dysfunction in cerebral artery, which is an important vessel to supply blood to brain 30. Of course, the underlying mechanism needs further investigations.

Pathological studies have shown markers of oxidative stress and inflammation in the damaged white matter associated with VCI 1. Supplementation of some agents, like the neurotrophic factor cerebrolysin, showed a modest cognitive improvement 31. Thus, we would expect that other mechanisms for vascular dementia such as oxidative stress and brain‐derived neurotrophic factors may also contribute to isocarbophos‐induced VCI.

A major limitation of this study is that we did not provide the direct evidence for vascular brain injury in rats. Our presented data only suggest the association between cerebral vascular dysfunction and cognitive impairment in isocarbophos‐treated rats. In this case, functional deficiencies of the cholinergic system, including a reduced level of ACh, have been observed in the cerebral cortex and hippocampus of patients 32. Inhibition of AChE is effective to improve cognitive performance in patients with AD 19, 33, implying that the AChE activity is increased in dementia. However, we observed that both AChE activity and ACh content were reduced by isocarbophos treatment in this study. We propose that this discrepancy may be explained by the reduction in ACh in vascular dementia. For example, both AChE activity and ACh content were decreased in rat BCCAO model of vascular dementia in this study. Though the cognitive symptoms of vascular dementia have common characteristics, infarction lesion is the main cause of cognitive dysfunction of BCCAO. However, we only observed the cerebral vascular dysfunction caused by isocarbophos. That is why we defined the model induced by isocarbophos as VCI but not vascular dementia. Generally, our results support the concept that chronic administration of isocarbophos induces cognitive impairment because of cerebral vascular dysfunction. In fact, overlap of AD neuropathology with cerebrovascular lesions is observed in up to 50% of cases of dementia 34.

Generally, this study demonstrates that organophosphate pesticides, such as isocarbophos, causes memory and learning impairment if rats are exposed to isocarbophos in long‐term. Mechanistically, the detrimental effects of isocarbophos on cognitive impairment are attributable to vascular dysfunction in the cerebral artery. These findings not only indicate that chronic exposure of isocarbophos is one of the risk factors contributing to VCI, but also suggest that the protective strategies on cerebrovascular function may prevent vascular dementia in patients exposed to organophosphate pesticides.

## Conflicts of interest

The authors confirm that there are no conflicts of interest.

## Author contribution

Peng Li designed and performed most of all experiments, and analysed all data. Ya‐Ling Yin, Mo‐Li Zhu, Guo‐Pin Pan, Fan‐Rong Zhao and Jun‐Xiu Lu partially did some experiments. Zhan Liu and Chang‐Ping Hu gave a lot of suggestions to revise the manuscript. Shuang‐Xi Wang convinced the whole project, wrote and revised the manuscript.

## Supporting information


**Figure S1** The scheme of animal experimental protocol.
**Table S1** Effects of isocarbophos on plasma acetylcholinesterase activity in rats.
**Table S2** Effects of isocarbophos on mirrors water maze test in rats.
**Table S3** The blood biochemical analysis and liver functions in rats.
**Data S1** Materials and methods.
