# Supplementary material for: Chronic administration of isocarbophos induces vascular cognitive impairment in rats
Source: J Cell Mol Med. 2016 Jan 28;20(4):731–9. doi: 10.1111/jcmm.12775 (PMC5125717; doi:10.1111/jcmm.12775)
Supplement: Supplementary file 1 — Figure S1 The scheme of animal experimental protocol. Table S1 Effects of isocarbophos on plasma acetylcholinesterase activity in rats. Table S2 Effects of isocarbophos on mirrors water maze test in rats. Table S3 The blood biochemical analysis and liver functions in rats. Data S1 Materials and methods. [file JCMM-20-731-s001.docx]

**Supplementary Information for**

**Chronic administration of isocarbophos induces vascular cognitive impairment in rats**

Peng Li^a,b^, Ya-Ling Yin^c,d^, Mo-Li Zhu^e^, Guo-Pin Pan^a^, Fan-Rong Zhao^e^, Jun-Xiu Lu^d^, Zhan Liu^f,*^, Shuang-Xi Wang^b,g,*^, Chang-Ping Hu^a,*^

^a^ Department of Pharmacology, Pharmaceutical College, Central South University, Changsha, China

^b^ College of Pharmacy, Xinxiang Medical University, Xinxiang, China

^c^ College of Basic Medical Sciences, Tongji Medical School, Huazhong University of Science and Technology, Wuhan, China

^d^ School of Basic Medical Sciences, Xinxiang Medical University, Xinxiang, China

^e^ Sanqun Medical College, Xinxiang Medical University, Xinxiang, China

^f^ Department of Clinical Nutrition, The Affiliated Hospital, Hunan Normal University, Changsha, China

^g^ The Key Laboratory of Cardiovascular Remodeling and Function Research, Qilu Hospital, Shandong University, Jinan, China

*Correspondence email: 346297715@qq.com

**Contents**

**1. Supplementary Materials and methods**

**2. Supplementary Figure S1**

**3. Supplementary Table S1-S3**

**Materials and methods**

**Animals**

Male Sprague-Dawley (SD) rats (4 weeks old, 180 ± 20 g) were provided by the Experimental Animal Center of Henan Province, license number: SYXK-Yu-005-0012). All rats were housed individually in cages at a room temperature of 18-22 °C with a 12-h light/dark cycle and given free access to food and water. The environment, such as cleanness, humidity, ventilation, air flow speed, air pressure, and automatic timer, was strictly controlled. This study was carried out in accordance with the recommendations in the Guide for the Care and Use of Laboratory Animals of the National Institutes of Health. The animal protocol was reviewed and approved by the Animal Care and Use Committee, University of Xinxiang Medical University.

**Materials**

Isocarbophos (Cat, LS93564) was purchased from Sha-Nong-Da Limited Company (Hubei, China). Phenylephrine (PE), and ACh were purchased from Sigma-Aldrich Company (Shanghai, China). Primary antibodies against CX43 (sc-13558), GFAP (sc-33673), Caspase-3 (sc-65496), DM1A (sc-32293), ICAM-1 (sc-390483) and VCAM-1 (sc-1504) were from Santa Cruz Company. The dilations of all antibodies in working solution were 1:1000 in Western blot and 1:100 in IHC or IFC, respectively. Commercial kits of AChE activity, and ACh quantification were obtained from Jian-Cheng Bioengineering Institute (Nanjing, China).

**In vivo experimental design**

All SD rats were randomly divided into three groups. Group 1: Saline-treated group; Group 2: isocarbophos-treated group; Group 3: Positive control group. Rats in group 1 intragastrically administrated with saline for 16 weeks (3 ml/kg, every other day). Rats in group 2 received gavage with isocarbophos (0.5 mg/kg, 3 ml/kg per two days) for 16 weeks. Rats in group 3 received the permanent ligation of bilateral common carotid artery occlusion (BCCAO). The details of procedures were presented in Supplementary Figure S1.

**Vascular dementia model by BCCAO**

The surgical procedure was performed as described previously [1]. Briefly, rats were anesthetized with a mixture of ketamine and xylazine (70:6 mg/kg, intramuscular injection) and placed under warm light (37°C). A midline incision was made to expose the bilateral common carotid arteries, and the exposed carotid arteries were tightly ligated with 4-0 silk sutures. After skin closure, rats were returned to their cages and normal feeding was resumed.

**Organ chamber**

Organ chamber was performed as described previously[2, 3]. Briefly, rings (3-5 mm in length) from rat middle cerebral artery were mounted in organ bath by 2 stainless hook in 5 ml Kreb’s solution (mM): NaCl 118.3, KCl 4.7, MgSO_4_1.2, KH_2_PO_4_1.2, CaCl_2_ 2.5, NaHCO_3_ 25.0, EDTA 0.026 and glucose 11.0 at 37°C, gassed with 95% O_2_ + 5% CO_2_, under a tension of 2 g, for 1 h equilibration period. During this period, the Kreb’s solution was changed every 15 min. After the equilibration, arteries were contracted with 60 mM KCl. After washing and another 30 minutes equilibration period, contractile response was evoked by PE (1 μM) to elicit reproducible responses. At the plateau of contraction, accumulative ACh (0.003 to 3 μM) was added into the organ bath to induce the endothelium dependent or independent relaxation. The relaxation induced by ACh at 3 μM was considered as the maximal effect (Emax).

**Step-Down Avoidance Test**

The latency time in the step-down avoidance test was determined to evaluate short-term memory as described previously[4]. The rats were trained in a step-down avoidance test on the tenth day after each stress exposure. Two hours after training, the latency time in each group was measured. The rats were placed on a 7X25X2.5 cm platform. The platform faced a 42X25 cm grid of parallel stainless steel bars that were 0.1 cm in caliber and spaced 1 cm apart. In the training sessions, the animals received a 0.5 mA scramble foot shock for 2 seconds immediately upon stepping down. The interval of time that elapsed between the rats stepping down and placing all four paws on the grid was defined as the latency time.

**Passive avoidance step-through task test**

This procedure was carried out as previously described, with some modifications[5]. The associative memory was assessed using passive avoidance step-through task. The apparatus consisted of a light and a dark compartment (20X20X40 cm) with an electrifiable grid floor. The two compartments were separated by a black partition with a rectangular doorway (8X8 cm). The lighted box was illuminated with a lamp (60 W positioned above the apparatus). The floor was constructed of stainless-steel grids 2 mm in diameter and at 8 mm intervals. Intermittent electric shock (50 Hz, 10 s, 0.5 mA intensity) was delivered to the grid floor of the dark compartment by an isolated stimulator.

An acquisition trial was performed for rats to habituate in both compartments freely for 3 min. During training, each rat was placed in light compartment; 10 s later the door between the compartments was opened and the latency to enter the dark compartment with all four paws was timed. Once the rat crossed with all four paws into the dark compartment, the door was closed and the 0.5 mA foot shock was administered for 10 s. The rat was removed from the apparatus and returned to its cage. If the rat waited for more than 100 s to cross to the dark compartment, they are excluded from the experiment.

**Morris Water Maze test**

Morris water maze test was used for learning and memory behavior assessment as described previously[6]. MWM was performed in a 180-cm diameter water pool and virtually divided into four quadrants. The pool was filled with water (22 ± 1°C). A colorless escape platform (10 cm in diameter) was submerged 2 cm beneath the water surface, located in a designated target quadrant. The maze was located in a quiet test room, surrounded by many visual cues outsides of the maze, which were visible from within the pool and could be used by the rats for spatial orientation. The pool was divided into four hypothetical quadrants. Data were acquired through a video camera connected to the computerized tracking system (Columbus Instruments, USA) fixed above the center of the pool.

At the last week of experiments, all rats were allowed to swim freely in the water maze for 5 min without platform. During training trial, the platform was positioned in the center of randomly selected quadrant and each rat was released always from the opposite quadrant facing toward the wall of the pool. The rat was allowed to find the platform spontaneously within 60 s and left on the platform for 5 s. If the rat failed to find the platform within 60 s it was gently directed toward the platform by the experimenter and left on the platform for 5 s. During the learning trial, rats were subjected to probe trials, for 30 s, on the same day at the end of the learning trial. Each rat received three to four learning session per day with 10 min interval between each trial. In each trial, the time taken by the rat to find the platform was recorded. Probe trial analysis reveals the index of memory in which the time spent in platform quadrant and number of times the rat crossed the platform area were noted.

**Determinations of AChE activity and ACh content**

AChE activity and ACh content in plasma or supernatant of hippocampus homogenates after centrifugation were determined by commercial assay kits according to the manufacturer’s instructions.

**Morphological analysis of vascular wall and hippocampus**

HE staining was performed to detect the global morphology of tissues as described previously[7]. The tissue specimens of hippocampus and cerebral artery from rats were fixed with 10% paraformaldehyde, followed by the preparation of the coronal sections, which were 5 μm thick, using a microtome (Leica RM2135, Nussloch, Germany). The paraffin-embedded brain sections were deparaffinized with xylene and rehydrated with ethanol at gradient concentrations of 100–70% (v/v), followed by washing with water. The sections were stained with hematoxylin and eosin and examined using light microscopy (Axio Observer A1 photomicroscope, Carl Zeiss, Germany).

For analysis of electron microscope, tissues were fixed with 2.5% glutaraldehyde at 4°C overnight. The tissues were then washed in phosphate buffered saline (0.1 M, pH7.0) three times followed by fixing in1% osmium tetroxide at room temperature (RT) for 1 h. Next, tissues were washed in water twice and stained in 2% uranyl acetate at RT for 1 h. Tissues were dehydrated by 70% ethanol for 15 min, 90% ethanol for 15 min, 100% ethanol twice for 15 min and finally 100% acetone once for 20 min. The samples were embedded into an embedding mixture (DDSA, MNA, DMP-30 and EPON812) and incubated at RT for 4-6 h. The samples were then sectioned into 70-90 nm and dyed with uranyl acetate and lead citrate. Finally, the slices were observed by TECNAI G20 transmission electron microscopy (Field Emission Inc.).

**Detections of ICAM-1 and VCAM1 by immunohistochemistry (IHC)**

As described previously with modifications[8], the cerebral artery was fixed in 4% paraformaldehyde overnight, and then processed, embedded in paraffin, and sectioned at 4 µm. The deparaffinized, rehydrated section was microwaved in citrate buffer for antigen retrieval. Sections were incubated in endogenous peroxidase (DAKO) and protein block buffer, and then with primary antibodies indicated overnight at 4°C. Slides were rinsed with washing buffer and incubated with labeled polymer-horseradish peroxidase-antimouse/antirabbit antibodies followed by DAB+ chromogen detection (DAKO). After final washes, sections were counterstained with hematoxylin. All positive staining was confirmed by ensuring that no staining occurred under the same conditions with the use of non-immune rabbit or mouse control IgG.

**Culture of primary neuron and astroglial cells from rats**

As described previously[9], male SD rats had their cerebral cortices aseptically dissected and meninges removed. During the dissection, the cortices were kept in Hank’s balanced salt solution (HBSS) containing 0.05% trypsin and 0.003% DNase and were kept at 37^0^C for 15 min. The tissue was then mechanically dissociated for 15 min using a Pasteur pipette and centrifuged at 400 g for 5 min. The pellet was resuspended in a solution of HBSS containing 40U papain/ml, 0.02% cysteine and 0.003% DNase and again gently mechanically dissociated for 15 min with a Pasteur pipette. After another centrifugation step (400 g, 5 min), the cells were resuspended in HBSS containing only DNase (0.003%) and left for decantation for 30–40 min. The supernatant was collected and centrifuged for 7 min (400 g). The cells from supernatant were resuspended in DMEM/F12 plus 10% fetal bovine serum, plated in 6- or 24-well plates pre-coated with poly-L-lysine and cultured at 37^0^C in a 95% air/5% CO_2_ incubator. The astroglial cells were seeded at 3-5 X 10^5^ cells/cm^2^.

Based on methods described previously[10], dissociated rat hippocampal primary neuron cultures were prepared. Briefly, a 25 mm glass cover slip (thickness, 0.08 mm) was glued over a 22 mm hole in the bottom of a 35 mm tissue culture dish using silicone sealant. Dissociated neuronal cultures from rat hippocampi at P1 and P2 were prepared. Neurons were plated onto prepared 35 mm tissue culture dishes at a density of 1X10^6^ cells per dish.

**Immunofluorescence (IFC)**

As described previously[11], cultured primary astroglial cells on sterile glass cover slips or hippocampal slices were rinsed by cold PBS and then fixed by incubation with 10% formalin in PBS for 10 minutes. Block tissues or cells by 5% BSA for 30 minutes. Incubate tissues or cells with primary antibody for 1 hour at room temperature or overnight at 4 ^o^C. After washing, incubate samples with fluoresence-conjugated secondary antibody for 45 minutes. Take picture under fluorescence microscope.

**Western blot**

Cells were homogenized on ice in cell-lysis buffer containing 20 mM Tris-HCl (pH 7.5), 150 mM NaCl, 1 mM Na_2_EDTA, 1 mM EGTA, 1% Triton, 2.5 mM sodium pyrophosphate, 1 mM beta-glycerophosphate, 1 mM Na_3_VO_4_, 1 µg/ml leupeptin, and 1 mM PMSF. Proteins were separated by SDS-PAGE, transferred to nitrocellulose membranes, and probed using specific antibodies. Band intensity (area × density) was measured by densitometry (model GS-700, Imaging Densitometer; Bio-Rad). Background intensity was subtracted from all calculated areas. The ration of caspase-3 to DM1A was used to represent the relative level of caspase-3, which was set up as 100% in control group.

**Statistical analysis**

All values are expressed as means ± SE. EC50 of ACh and correction between AChE activity in plasma and hippocampus were calculated by using GraphPad Prism V5.0 Software. Data were analyzed using a one-way or two-way ANOVA followed by Newman-Student’s t-test. For statistical comparison between two groups, we used an unpaired Student's t-test. All statistical analysis was performed with SPSS 13.0 software.

**References**

1. **Browne RO, Moyal-Segal LB, Zumsteg D*, et al.*** Coding region paraoxonase polymorphisms dictate accentuated neuronal reactions in chronic, sub-threshold pesticide exposure. *FASEB J*. 2006; 20: 1733-5.

2. **Yang XH, Li P, Yin YL*, et al.*** Rosiglitazone via PPARgamma-dependent suppression of oxidative stress attenuates endothelial dysfunction in rats fed homocysteine thiolactone. *J Cell Mol Med*. 2015; 19: 826-35.

3. **Wang S, Xu J, Song P*, et al.*** In vivo activation of AMP-activated protein kinase attenuates diabetes-enhanced degradation of GTP cyclohydrolase I. *Diabetes*. 2009; 58: 1893-901.

4. **Li TJ, Qiu Y, Yang PY*, et al.*** Timosaponin B-II improves memory and learning dysfunction induced by cerebral ischemia in rats. *Neurosci Lett*. 2007; 421: 147-51.

5. **Kumaran D, Udayabanu M, Kumar M*, et al.*** Involvement of angiotensin converting enzyme in cerebral hypoperfusion induced anterograde memory impairment and cholinergic dysfunction in rats. *Neuroscience*. 2008; 155: 626-39.

6. **Vorhees CV, Williams MT.** Morris water maze: procedures for assessing spatial and related forms of learning and memory. *Nat Protoc*. 2006; 1: 848-58.

7. **Wang S, Xu J, Song P*, et al.*** Acute inhibition of guanosine triphosphate cyclohydrolase 1 uncouples endothelial nitric oxide synthase and elevates blood pressure. *Hypertension*. 2008; 52: 484-90.

8. **Wang S, Zhang M, Liang B*, et al.*** AMPKalpha2 deletion causes aberrant expression and activation of NAD(P)H oxidase and consequent endothelial dysfunction in vivo: role of 26S proteasomes. *Circ Res*. 2010; 106: 1117-28.

9. **Souza DG, Bellaver B, Souza DO, Quincozes-Santos A.** Characterization of adult rat astrocyte cultures. *PLoS One*. 2013; 8: e60282.

10. **Hoover BR, Reed MN, Su J*, et al.*** Tau mislocalization to dendritic spines mediates synaptic dysfunction independently of neurodegeneration. *Neuron*. 2010; 68: 1067-81.

11. **Wang S, Zhang C, Zhang M*, et al.*** Activation of AMP-activated protein kinase alpha2 by nicotine instigates formation of abdominal aortic aneurysms in mice in vivo. *Nat Med*. 2012; 18: 902-10.

**
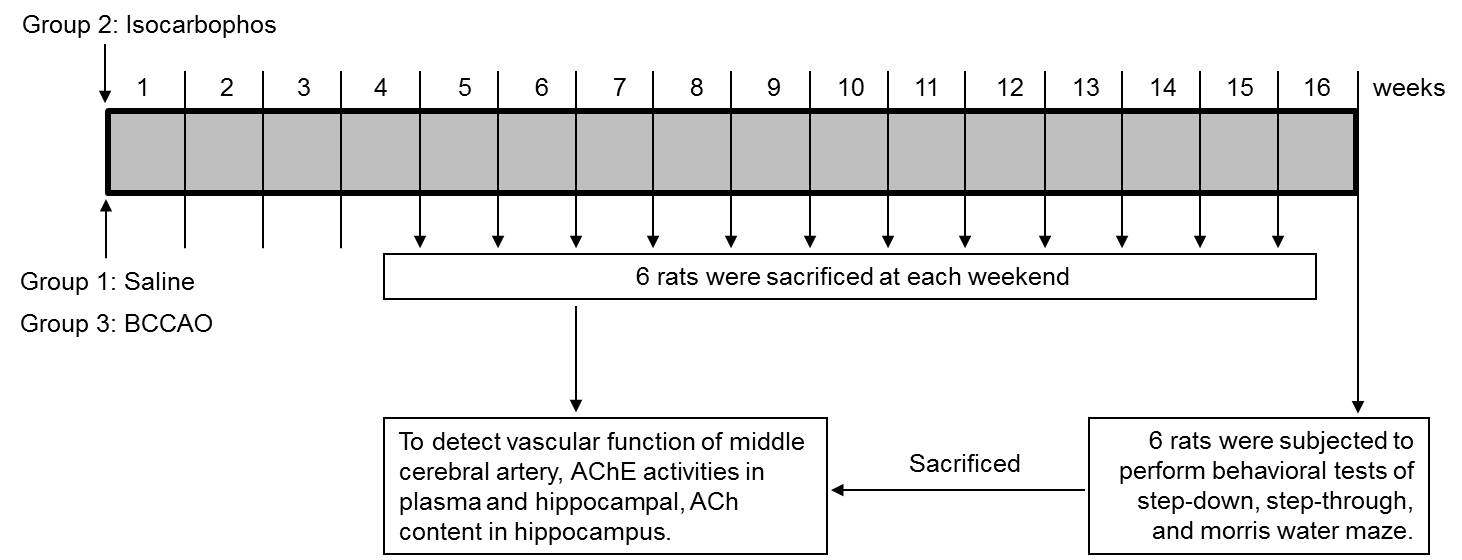
**

**Supplementary Figure S1. The scheme of animal experimental protocol.** After 4-week isocarbophos administration, 6 rats were killed at each weekend from the 4^th^-16^th^ week to test AChE activities in plasma and hippocampal, ACh content in hippocampus and vascular function of middle cerebral artery. At the end of experiment, 6 rats were subjected to perform behavioral tests of step-down, step-through, and morris water maze and morphological analysis of middle and posterior cerebral artery or hippocampus were observed under light microscopy and electron microscopy.

**Supplementary Table S1. Effects of isocarbophos on plasma acetylcholinesterase activity in rats**

| **Groups** | **4 W** | **5 W** | **6 W** | **7 W** | **8 W** | **9 W** | **10 W** | **11 W** | **12 W** | **13 W** | **14 W** | **15 W** | **16 W** |
| --- | --- | --- | --- | --- | --- | --- | --- | --- | --- | --- | --- | --- | --- |
| Saline | 37.72±7.43 | 35.29±5.44 | 36.29±4.59 | 35.69±6.72 | 34.75±7.51 | 37.42±4.52 | 35.18±5.49 | 38.42±6.41 | 36.46±5.64 | 36.57±6.08 | 37.42±7.41 | 36.59±5.49 | 37.52±6.37 |
| Isocarb-ophos | 34.94±6.22 | 37.73±7.16 | 38.44±6.51 | 38.26±6.37 | 35.49±8.32 | 38.64±6.57 | 37.65±6.33 | 37.42±4.52 | 37.55±5.92 | 39.54±6.54 | 36.51±4.22 | 38.42±5.49 | 36.84±6.51 |
| Positive control | 36.27±4.69 | 38.62±6.31 | 35.46±5.66 | 37.22±4.97 | 36.16±6.44 | 36.51±6.39 | 36.54±4.81 | 36.51±6.47 | 38.46±8.63 | 37.45±5.47 | 36.29±6.55 | 34.28±6.41 | 36.84±5.84 |

After isocarbophos treatment, blood was harvested at indicated time-point. Plasma acetylcholinesterase activity was assayed. Data are expressed as means ± SE. N is 6 in each group.

**Supplementary Table S2. Effects of isocarbophos on mirrors water maze test in rats**

| **Groups** | **Saline** | **Isocarbophos** | **Positive control** |
| --- | --- | --- | --- |
| Swimming distance in quadrant (m) | 2.45±0.58 | 1.29±0.35 (*P*=0.00127) | 1.35±0.42 (*P*=0.00224) |
| Swimming distance in quadrant/Swimming distance | 0.48±0.17 | 0.35±0.08 (*P*=0.02182) | 0.32±0.09 (*P*=0.00672) |
| Swimming time in quadrant (s) | 31.26±6.39 | 19.58±3.21 (*P*=0.00069) | 18.25±4.69 (*P*=0.00037) |
| Swimming time in quadrant/Swimming time | 1.13±0.09 | 0.59±0.08 (*P*=0.00208) | 0.52±0.06 (*P*=0.00085) |
| Swimming time out of quadrant (s) | 28.78±5.69 | 41.68±6.43 (*P*=0.00096) | 43.33±7.26 (*P*=0.00174) |
| Swimming distance out of quadrant (m) | 2.09±0.61 | 2.86±0.68 (*P*=0.03627) | 2.95±0.98 (*P*=0.00691) |
| Number of escape | 6.95±2.11 | 3.64±0.89 (*P*=0.00106) | 3.88±0.72 (*P*=0.00073) |
| Swimming distance (m) | 4.23±1.21 | 4.32±0.99 | 4.09±1.01 |
| Swimming speed in quadrant (mm/s) | 69.29±10.23 | 65.29±8.12 | 67.48±11.98 |
| Swimming speed out quadrant (mm/s) | 68.44±9.77 | 67.29±12.54 | 69.45±10.28 |
| Average Swimming speed (mm/s) | 68.98±6.91 | 69.84±7.12 | 69.84±7.12 |

Before the end of experiment, mirrors water maze test was performed in all rats. Data are expressed as means ± SE. N is 6 in each group. *P* value means Isocarbophos or Positive control VS saline. All results were analyzed using a one-way ANOVA followed by Newman-Student’s t-test.

**Supplementary Table S3. The blood biochemical analysis and liver functions in rats**

| **Groups** | **Saline** | **Isocarbophos** |
| --- | --- | --- |
| K^+^ (mM) | 4.01±0.57 | 4.29±0.55 |
| Na^+^ (mM) | 143±24 | 135±28 |
| Cl^-^ (mM) | 105±13 | 101±21 |
| Ca^2+^ (mM) | 2.96±0.4 | 3.54±0.58 |
| Mg^2+^ (mM) | 0.91±0.11 | 0.88±0.09 |
| P (mM) | 1.02±0.17 | 1.60±0.27 |
| BUN (mM) | 5.3±0.64 | 5.64±0.80 |
| Creatinine (μM) | 73±9 | 84±12 |
| Glucose (mM) | 6.87±0.75 | 7.99±0.82 |
| Triglycerin (mM) | 1.17±0.23 | 0.92±0.32 |
| Cholesterol (mM) | 5.32±0.69 | 4.17±1.08 |
| Homocysteine (mM) | 8.75±2.31 | 16.5±1.87  (*P*=0.00054) |
| Glutamic-pyruvic Transaminase (U/L) | 28±4 | 33±5 |
| Glutamic-oxalacetic Transaminase (U/L) | 21±3 | 30±6 |
| Total Protein (g/L) | 70.9±9.4 | 78.6±105 |
| Albumin (g/L) | 47.3±2.11 | 54.0±6.89 |
| Globulin (g/L) | 23.6±0.37 | 24.6±3.19 |
| Total Bilirubin (μM) | 12.2±1.58 | 15.9±2.7 |
| Direct Bilirubin (μM) | 3.5±0.51 | 5.9±0.5 |
| Indirect Bilirubin (μM) | 8.7±0.90 | 10.0±1.2 |

At the 16^th^ weekend after isocarbophos treatment, rats were sacrifice and blood was collected to perform blood biochemical analysis and measure liver functions. Data are expressed as means ± SE. N is 6 in each group. An unpaired Student's t-test was used for statistical analysis.
